# Cloning and expressing a highly functional and substrate specific farnesoic acid o-methyltransferase from the Asian citrus psyllid (*Diaphorina citri* Kuwayama)

**DOI:** 10.1016/j.fob.2015.03.012

**Published:** 2015-03-28

**Authors:** Evelien Van Ekert, Robert G. Shatters, Pierre Rougé, Charles A. Powell, Guy Smagghe, Dov Borovsky

**Affiliations:** a USDA-ARS, Ft. Pierce, FL 34945, USA; b Université de Toulouse, UPS, Institut de Recherche pour le Développement (IRD), UMR 152 Pharma-Dev, Université Toulouse 3, Faculté des Sciences Pharmaceutiques, F-31062 Toulouse cedex 09, France; c Indian River Research and Education Center, University of Florida, FL 34945, USA; d Universiteit Gent, Gent 9000, Belgium

**Keywords:** FA, farnesoic acid, FA-o-MT, farnesoic acid o-methyltransferase, *fmt*D, *Diaphorina citri* farnesoic acid o-methyltransferase gene, GAPDH, glyceraldehyde 3-phosphate dehydrogenase, JH, juvenile hormone, JHA, juvenile hormone acid, JHAMT, juvenile hormone acid methyltransferase, *jmtD*, juvenile hormone acid methyl transferase ortholog gene of *Diaphorina citri*, MF, methyl farnesoate, MMLV, Moloney murine leukemia virus, RP, reversed phase, SAM, S-adenosyl methionine, SAM-MT, S-adenosyl methionine-dependent methyltransferase, *Diaphorina citri*, Juvenile hormone acid methyltransferase, Farnesoic acid methyltransferase, 3D modeling, Gene expression

## Abstract

The Asian citrus psyllid, *Diaphorina citri*, transmits a phloem-limited bacterium, *Candidatus* ‘Liberibacter’ asiaticus that causes citrus greening disease. Because juvenile hormone (JH) plays an important role in adult and nymphal development, we studied the final steps in JH biosynthesis in *D. citri*. A putative JH acid methyltransferase ortholog gene (*jmt*D) and its cognate cDNA were identified by searching *D. citri* genome database. Expression analysis shows expression in all life stages. In adults, it is expressed in the head-thorax, (containing the corpora allata), and the abdomen (containing ovaries and male accessory glands). A 3D protein model identified the catalytic groove with catalytically active amino acids and the S-adenosyl methionine (SAM)-binding loop. The cDNA was expressed in *Escherichia coli* cells and the purified enzyme showed high preference for farnesoic acid (FA) and homoFA (*k_cat_* of 0.752 × 10^−3^ and 0.217 × 10^−3^ s^−1^, respectively) as compared to JH acid I (JHA I) (*cis/trans/cis*; 2*Z*, 6*E*, 10*cis*), JHA III (2*E*, 6*E*, *10cis*), and JHA I (*trans*/*cis*/*cis*; 2*E*, 2*Z*, 10*cis*) (*k_cat_* of 0.081 × 10^−3^, 0.013 × 10^−3^, and 0.003 × 10^−3^ s^−1^, respectively). This suggests that this ortholog is a *Dc*FA-o-methyl transferase gene (*fmt*D), not a *jmt*D, and that JH biosynthesis in *D. citri* proceeds from FA to JH III through methyl farnesoate (MF). *Dc*FA-o-MT does not require Ca^2+^, Mg^2+^ or Zn^2+^, however, Zn^2+^ (1 mM) completely inhibits the enzyme probably by binding H115 at the active groove. This represents the first purified FA-o-MT from Hemiptera with preferred biological activity for FA and not JHA.

## Highlights

• A putative JH acid methyltransferase ortholog gene (*jmtD*) and its cognate cDNA were identified in *D. citri*.
• This gene is expressed in all life stages in *D. citri*.
• The purified enzyme expressed from cDNA in *E. coli* showed high preference for farnesoic acid (FA) and homoFA.
• This suggests that this ortholog is a *Dc*FA-o-methyl transferase gene (*fmt*D), and not *jmt*D.
• First purified FA-o-MT from any insect order with preferred biological activity for FA and not JHA.

## Graphical abstract

**Figure:**
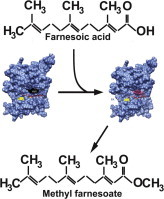

## 1 Introduction

*Diaphorina citri* is a phloem-feeding citrus hemipteran pest causing major economic damage to citrus by salivary transfer of *Candidatus* Liberibacter bacterium into the citrus phloem [1] which causes citrus greening disease or huanglongbing (HLB) [2]. Symptoms of the disease include yellowing of the leaves, stunted growth, bitter fruit followed by premature defoliation, dieback of twigs, and decay of feeder rootlets and lateral roots [2,3]. Currently, there is no cure and the infected trees ultimately die [3]. Thus, the best strategy for disease management is to remove infected trees in combination with aggressive psyllid control. Concerns about long-term heavy reliance on broad-spectrum insecticides include residue accumulation in fruit, environmental impact, and ultimate appearance of psyllid insecticide resistance. Therefore, developing target specific biological insecticides with minimal environmental impact is urgently needed.

Juvenile hormone (JH) participates in many physiological processes in insects and external application of JH mimics (e.g., methoprene and pyriproxyfen) often cause detrimental effects on molting, metamorphosis, and reproduction of insects [4,5]. In the laboratory, pyriproxyfen causes ovicidal and nymphicidal effects reducing fecundity in *D. citri* [5]. Since these insecticides are not species-specific they may affect beneficial insects, when sprayed in citrus groves. Therefore, we have established a research project to determine if a more species-specific control strategy can be developed by targeting this process. Since the biosynthetic pathway of JH is tightly regulated in insects [6], targeting specific enzymes in the JH pathway using species-specific molecules like dsRNA moieties could control diverse agricultural and medical important insects [7]. There are two routes for the last two biosynthetic steps in JH III biosynthesis. First, JH III precursor, farnesoic acid (FA), can be converted into JHA III by cytochrome P-450 linked epoxidase and then methylated into JH III by juvenile hormone methyltransferase (JHAMT) using SAM. Second, FA can be first converted by farnesoic acid-o-methyltransferase (FA-o-MT) using SAM, into methyl farnesoate (MF) following epoxidation with cytochrome P-450 epoxidase into JH III [8,9]. JHAMT orthologs of *Samia cynthia ricini* and *Aedes aegypti* were extensively characterized by Sheng et al. [10] and Van Ekert et al. [11], respectively. Van Ekert et al. [11] determined, for the first time, the *K_m_*¸ *V_max_*, and *k_cat_ of A. aegypti* (*Aea*)JHAMT showing that the enzyme prefers to methylate JHA III and not FA proving that *Aea*JHAMT is the ultimate enzyme in the JH biosynthetic pathway. In hemipterans, on the other hand, no information on the JH biosynthetic pathway exists. Because hemipterans comprise many important agricultural pest insects, characterization of *D. citri* SAM-MT may help developing control strategies for hemipterans if JH biosynthetic pathway could be blocked with enzyme specific inhibitors or dsRNA [7]. While JHAMT orthologs in *Bombyx mori*, *Drosophila melanogaster*, *Tribolium castaneum*, and *A. aegypti* convert FA into MF at a substantially lower rate [11–15], to date, no JHAMT ortholog is known to prefer FA over JHA III as its primary substrate except in *B. mori* where FA is preferred over JHA III [58]. It was suggested that FA-o-MT is a distinctly different enzyme that methylates FA using SAM and converts it into MF in Dictyoptera and Orthoptera [8]. The first identification, cloning and expression of an active FA-o-MT was done in the crustacean sand shrimp, *Metapenaeus ensis* De Haan (Decapoda: Penaeidae). In this organism, JH III is not synthesized and MF is important in development and reproduction [16]. Following this report and subsequent studies in other Crustacea (*Litopenaeus vannamei*, *Homarus americanus*, *Cancer pagurus*) and insect species (*Ceratitis capitata*, *Nilaparvata lugens* and *Melipona scutellaris*) did not verify this activity [17–22]. FA-o-MT orthologs were found in the genomes of *D. melanogaster* [23,24] and the desert locust, *Schistocerca gregaria* [25], however, these reports show that the identified FA-o-MT orthologs are not involved in JH biosynthesis. Furthermore, all the reported orthologs of these FA-o-MT sequences (identified by homology to a characterized crustacean FA-o-MT) lack a SAM consensus binding region [26]. It is now generally regarded that insect JHAMTs can utilize both FA and JHA as substrates [8], however, the affinity of the enzyme is much higher to JHA III than to FA as was shown by Van Ekert et al. [11] in *A. aegypti* using a purified JHAMT. Despite the ambiguity in identifying which enzyme synthesizes MF in insects, it is clear that MF can be produced and secreted by the corpora allata (CA) [27] suggesting that MF may have a biological role in *D. melanogaster* and *A. aegypti*. In Hemiptera, however, the importance of MF and its role in JH biosynthesis is even less clear. On the other hand the role of JH bisepoxide was first observed in heteropterans [59]. Here we report the identification of a *D. citri Dc*FA-o-MT containing SAM-MT binding site sequence with high affinity for FA and low affinity for JHA III.

## 2 Results

### 2.1 Sequence analysis of *fmt*D

**Fig. 1:**
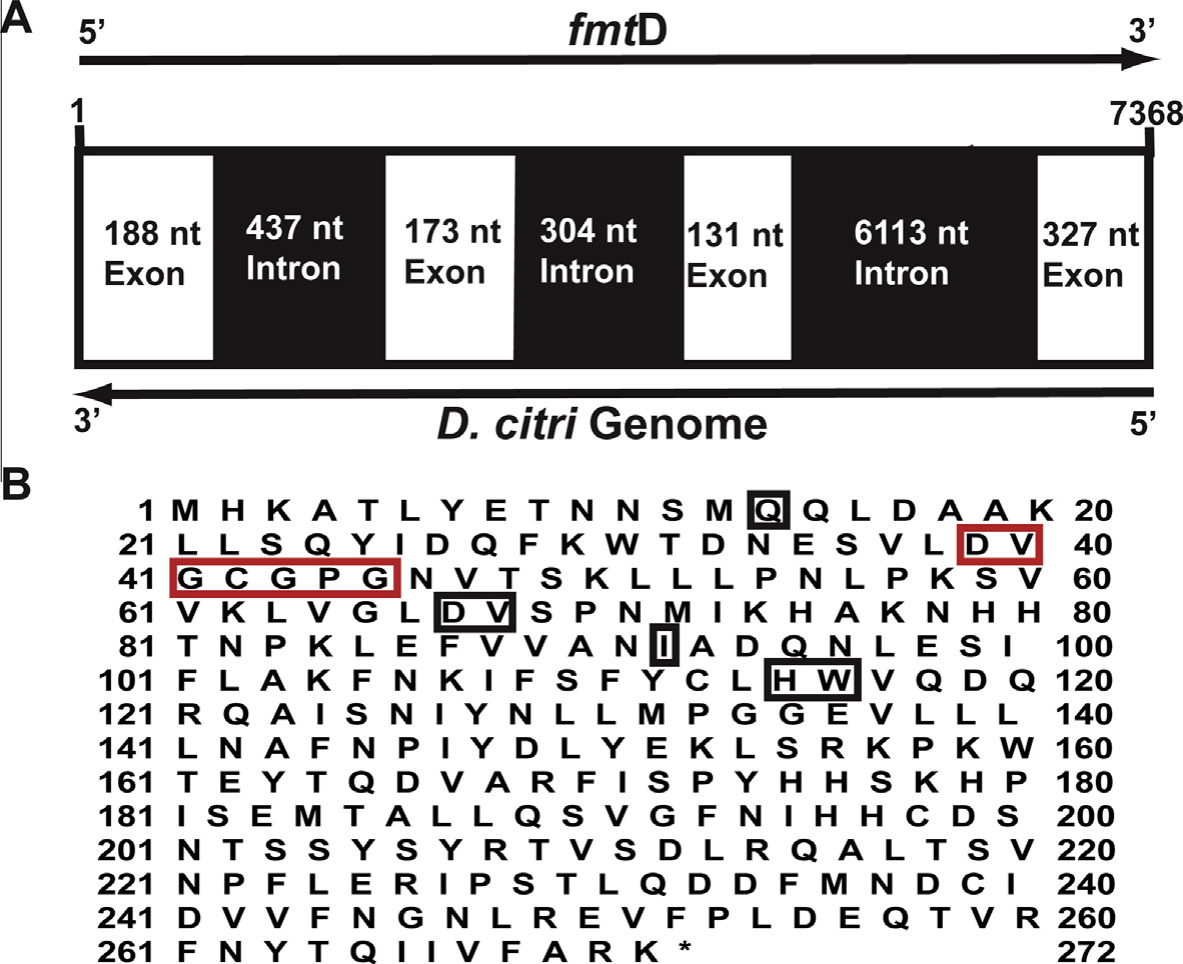
(A) Diagram representing the full-length *fmt*D sequence (7368 bp) including 4 exons (white color) and 3 introns (black color). The *fmt*D sequence (5′–3′) runs opposite to the *D. citri* genome. (B) Translated amino-acid sequence of the *fmt*D ORF (272 amino acids) including SAM-MT motif (red box) and amino acids that are in contact with the substrate and SAM at the active groove (black boxes) including H115 and W116.

**Fig. 2:**
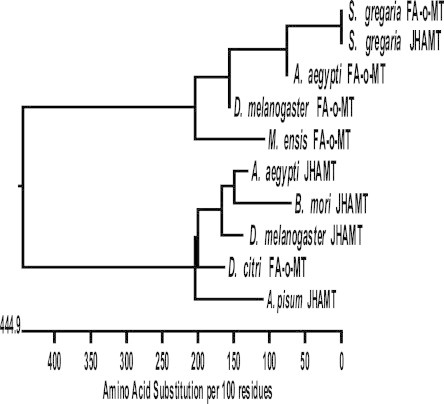
ClustalW tree representation of *D. citri* FA-o-MT in relationship to JHAMT orthologs of *A. pisum*[26]*¸D. melanogaster*[14], *B. mori*[15], *A. aegypti*[11], *S. gregaria*[25] and FA-o-MT orthologs of *M. ensis*[16]¸ *D. melanogaster*[23], *A. aegypti*[11], and *S. gregaria*[25].

The ORF of *fmt*D, was obtained by exploring the *D. citri* transcriptome and genome sequences using bioinformatics and RT-PCR amplification with gene-specific primers and the cDNA sequence was deposited in GenBank (accession number KM212828). The full-length *fmt*D ORF sequence was blasted against the available genome data (http://psyllid.org/download) identifying *fmt*D (7368 bp long), containing 3 introns (437, 304 and 6113 nt, each) and 4 exons (188, 173, 131 and 327 nt, each) encoding 272 amino acids *Dc*FA-o-MT that is directionally opposite (5′–3′) to the *D. citri* genomic scaffold 5.1 (Fig. 1). ClustalW alignment followed by phylogenetic analysis of the translated amino acid sequence shows sequence similarities between *Dc*FA-o-MT and the JHAMT orthologs of *A. aegypti*, *D. melanogaster*, *B. mori*, and *Acyrthosiphon pisum* including the conserved glycine-rich motif (DVGCGPG) involved in SAM-binding, and the amino acids D38, D67, V68, I92, Q14, H115 and W116 found in the JHAMT orthologs of *A. aegypti*, *B. mori*, *D. melanogaster*, *S. cynthia ricini*, *S. gregaria* and *Apis mellifera* (Figs. 1B and 2) [10–12,14,15,25,26]. ClustalW and phylogenetic analyses of *Dc*FA-o-MT shows that the enzyme is much more similar to the JHAMT orthologs of *A. pisum*, *D. melanogaster*, *B. mori* and *A. aegypti* than to the previously reported FA-o-MT-like sequences of *M. ensis*, *A. aegypti*, and *S. gregaria* that lack a SAM binding site sequence (Fig. 2).

### 2.2 Bacterial expression and enzyme purification

**Fig. 3:**
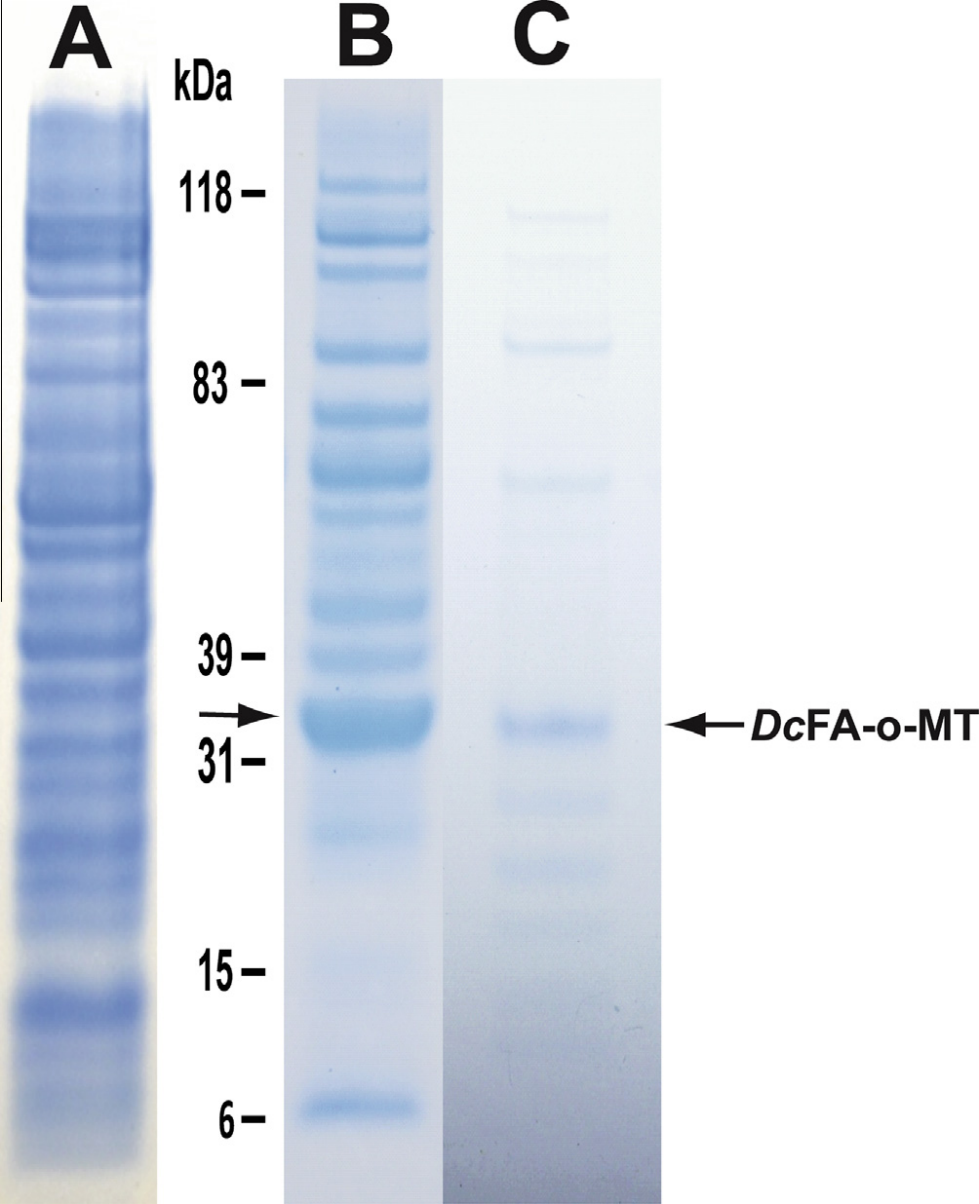
SDS PAGE of *Dc*FA-o-MT. Lane A, Crude extract (50 μg) of *E. coli* cells that were cloned with a plasmid that does not carry *Dc*FA-o-MT (control). Lane B, crude bacterial extract (50 μg) of *E. coli* cells that were cloned with a plasmid carrying *Dc*FA-o-MT. Lane C, purified *Dc*FA-o-MT (20 μg) after Ni-affinity chromatography. Arrow points to the location of *Dc*FA-o-MT (*M_r_* 31.2) protein band in lanes B and C which is missing in lane A (control).

*Dc*FA-o-MT was expressed in *Escherichia coli*, and purified by nickel affinity chromatography. The bacterial cells after IPTG induction and before column purification expressed a distinct protein band at *M_r_* 31 kDa after an aliquot (50 μg) was analyzed by SDS PAGE, whereas *E. coli* cells that were induced with IPTG and transformed with a plasmid that did not carry *Dc*FA-o-MT and analyzed by SDS PAGE (50 μg) did not show this protein band (Fig. 3 lanes A, B). After breaking the bacterial cells (500 mL) and centrifugation, 53.5 mg of the crude extracted proteins were adsorbed onto a Ni–NTA affinity column and 8.7 mg of the purified *Dc*FA-o-MT was recovered. The purified protein (20 μg) was analyzed by SDS PAGE identifying a major protein band corresponding with the expected recombinant *Dc*FA-o-MT (*M_r_* 31 kDa) (Fig. 3 lane C as compared with lane B) and the enzyme at this stage of purification is about 80% pure. Several minor bands below 31 kDa may indicate break down of the protein during the affinity purification steps. Aliquots (0.2 mL) were stored in the presence of 20 mM β–ME and glycerol (50%) and the purified enzyme at this stage is stable when stored at −20 °C.

### 2.3 Molecular modeling

**Fig. 4:**
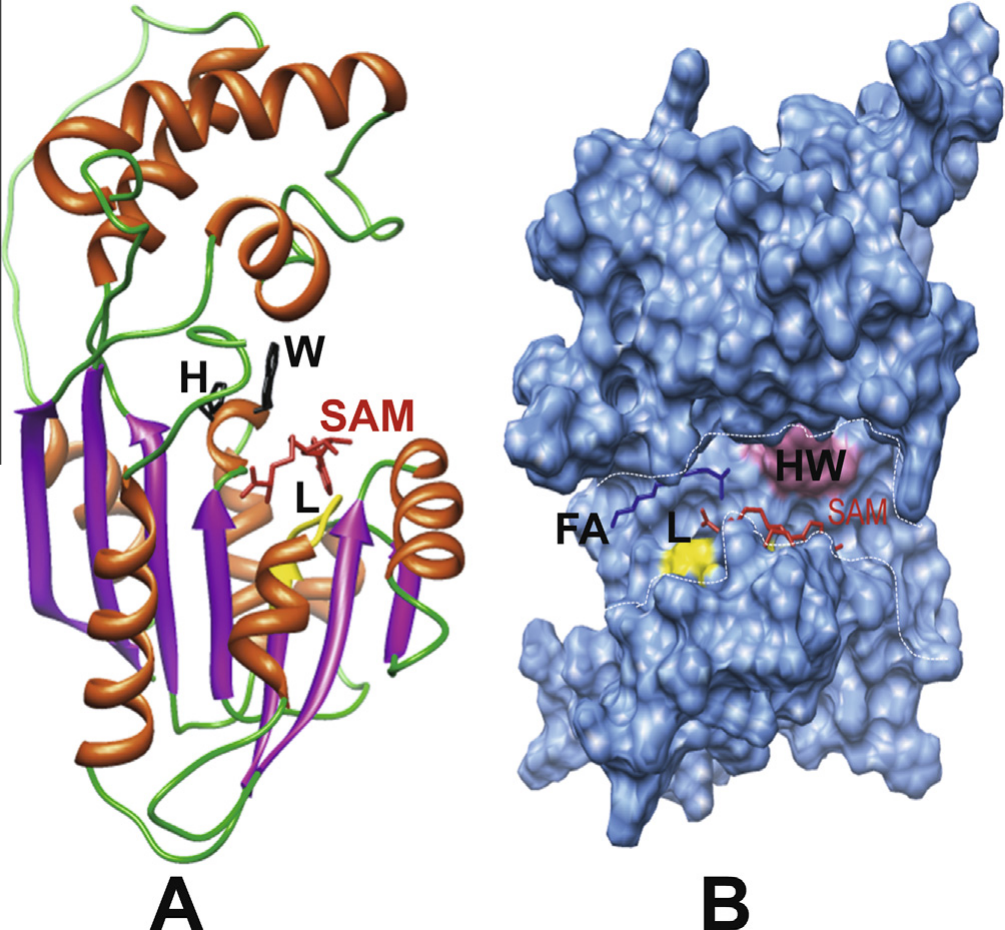
(A) 3D side view of a ribbon diagram representation of *Dc*FA-o-MT, with SAM molecule (red) docked into the catalytic groove. The loop DVGCGPG (L, yellow color) allows SAM to bind into the groove including the active residues H115 and W116 (black color), respectively. (B) Front view of the molecular contour surface of *Dc*FA-o-MT, showing SAM (red color) and the substrate, FA, (blue color) docked into the active catalytic groove (marked by white dashes) The SAM-binding loop, L, (yellow color) and the active residues H115 and W116 (pink color) are also shown.

A hybrid 3D model was built with the YASARA structure program. PROCHECK revealed that all of *Dc*FA-o-MT amino acid residues were correctly assigned in the allowed regions of the Ramachandran plot, except for two residues, W31 and K58, which occur in the non-allowed region of the plot. These residues are located at the loop region that connects α-helices. ANOLEA showed that only 7 out of 272 *Dc*FA-o-MT residues exhibited an energy level over the threshold value. The hybrid 3D model exhibits the canonical structural organization of the SAM-MTs, made of a first globular domain containing extended β-sheets characteristic of all other SAM-MTs [35], and a second α-helical cap domain (Fig. 4A). The two domains form a long and large enough groove allowing the accommodation of SAM as a methyl donor for FA (Fig. 4A and B). The loop containing a SAM-binding region (DVGCGPG) is found at the bottom of the groove (Figs. 1 and 4), where D can be either E or D, VG can be several hydrophobic amino acids, while C, G and P are generally C, S or T and the last G is highly conserved [60]. The groove is sufficiently extended to accommodate FA or JHA III (not shown) at the opposite side of the SAM-binding cavity by molecular docking. FA binds by hydrophobic interactions to the hydrophobic amino acid residues bordering the groove (Fig. 4B). The catalytically active H116, associated with the conserved W115, are located at the center of the groove and positioned in such a way that the carboxylic group of the docked FA becomes correctly aligned allowing methyl group transfer from SAM (Fig. 4B). Our modeling and docking predicts that both FA and JHA III can be methylated by *Dc*FA-o-MT.

### 2.4 Kinetic studies

**Fig. 5:**
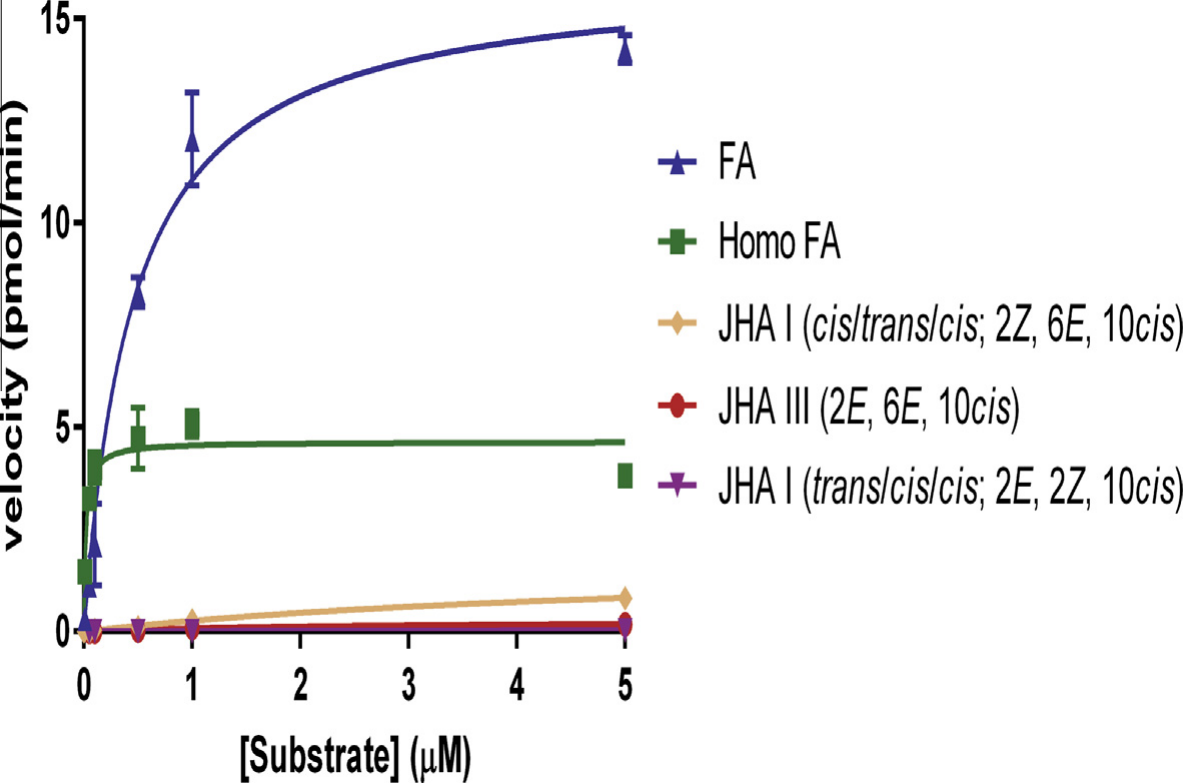
Substrate specificity of *Dc*FA-o-MT (357 pmol) in the presence of a saturating SAM concentration (10 μM) and different substrates. Reaction velocities (pmol/min) are plotted against increasing substrate concentrations of: FA (blue), homoFA (green), JHA I (*cis/trans/cis*; 2*Z*, 6*E*, 10*cis*) (orange), JHA I (*trans/cis/cis*; 2*E*, 6*Z*, 10*cis*) (purple) and JHA III (2*E*, 6*E*, 10*cis*) (red). Hyperbolic curves were fitted using GraphPad Prism v5.0.

To determine the enzyme’s substrate specificity, it was incubated with different substrates (FA, homoFA, JHA III (2*E*, 6*E*, 10*cis*), JHA I (*cis/trans/cis*; 2*Z*, 6*E*, 10*cis*) and JHA I (*trans/cis/cis*; 2*E*, 6*Z*, 10*cis*) and incubations for up to 45 min were linear (*r*^2^ > 0.96 and runs test *p* = 1). Based on these observations, a 30-min incubation period and SAM saturating concentration (10 μM) were used in all the kinetic studies to determine the *K_m_*, *V_max_* and *k_cat_* for different substrates. Plotting the reaction velocity (pmol/30 min) against increasing substrate concentrations (μM), shows that FA is the best substrate (Fig. 5, Table 2) with the highest turnover number (*k_cat_* = 0.75 ± 0.03 × 10^−3^ s^−1^) that is 3.5, 9.3, 57.8 and 251- fold higher than homoFA, JHA I (*cis/trans/cis*; 2*Z*, 6*E*, 10*cis*), JHAIII and JHA I (*trans/cis/cis*; 2*E*, 6*Z*, 10*cis*), respectively. The calculated *K_m_* of FA (0.46 ± 0.05 μM) is 23.7-fold higher than homoFA but 12.3-fold lower than JHA I (*cis/trans/cis*; 2*Z*, 6*E*, 10*cis*), 5.5-fold lower than JHA III and 100-fold lower than JHA I (*trans/cis/cis*; *2E*, 6*Z*, 10*cis*). These results indicate that the enzyme exhibits high affinities to FA and homoFA which can be converted into methyl farnesoic acid and methyl homofarnesoate, respectively and then epoxidized into JHIII and JHI by cytochrome P-450 epoxidase, respectively. Even though homoFA shows higher affinity to the enzyme and 6.6-fold higher catalytic activity (*k_cat_*/*K_m_*) the maximal rate (*V_max_*) of converting FA into MF is 3.5-fold faster than converting homoFA into methylhomofarnesoic acid indicating that chirality at the active groove is important for efficient methylation (Fig 5, Table 2). On the other hand, JHA I (*cis/trans/cis*; 2*Z*, 6*E*, 10*cis*), JHA III and JHA I (*trans/cis/cis*; 2*E*, 6*Z*, 10*cis*) are poor substrates (Fig. 5, Table 2), indicating that the enzyme prefers FA over JHA III or JHA I and thus, it is a FA-o-MT and not JHAMT. JHA I bisepoxide and JHA III bisepoxide could not be converted into their respective methyl esters indicating that the additional epoxide ring interferes with proper alignment at the active groove of *Dc*FA-o-MT.

### 2.5 Effect of metals

To determine if Mg^2+^, Ca^2+^ or Zn^2+^ are needed for enzymatic activity, increasing concentrations of MgCl_2_, CaCl_2_, and ZnSO_4_·H_2_O (1–10 mM) were incubated with *Dc*FA-o-MT. Addition of MgCl_2_ (5 mM) and CaCl_2_ (10 mM) significantly reduced the enzyme activity only by 1.2 and 1.5-fold, respectively (*p* < 0.001). However, 1 mM ZnSO_4_·H_2_O reduced the enzyme activity by 200-fold (Fig. 7) (*p* < 0.001), Indicating that these metal ions are not needed for stability. The highest inhibition observed with the addition of Zn^2+^ (1 mM) indicates that it probably binds to H116 at the active groove preventing catalysis (Fig. 4A).

### 2.6 Gene expression

Expression of *fmt*D transcript was followed during the different life stages of *D. citri* in several tissues. The *fmt*D transcript level in laid eggs (24 h old) is high; therefore, transcript expression at that stage was used to compare *fmt*D transcripts at different nymphal stages. Using ΔΔ*C_T_* determinations and ANOVA significant difference between nymphal stages was found (*F* = 8.48, df = 5, *p* = 0.0003). Tukey’s post hoc test shows that *fmt*D transcript level in laid eggs is significantly higher than in all the five instars (*p* < 0.01). No significant difference, however, was observed between the *fmt*D mRNA level at different nymphal stages (*p* > 0.05) (Fig. 7). *D. citri* female and male adults express *fmt*D in the head-thorax region, containing the corpora allata, and in the abdominal region, containing the female ovaries or the male accessory glands (Fig. 8A–D). To find out if female *D. citri* with green and brown abdomens synthesize different amounts of *fmt*D transcripts, head-thoraces were analyzed and show that there is a significant difference between the green and brown groups (*F* = 2.92, df = 7, *p* = 0.04). Tukey’s post hoc test shows that *fmt*D transcript is significantly higher in green female head-thoraces at day 6 as compared with day 1, and day 3, after eclosion (*p* < 0.05) (Fig. 8A). These females also exhibit significantly higher level of *fmt*D transcript at day 8 as compared with day 1 after eclosion (*p* < 0.05). No significant differences were observed in brown female head-thoraces (*p* > 0.05) (Fig. 8A). An upward trend in expression over time was observed in both green and brown females, but high variation between the 3 different biological replicates at day 6 and day 8, makes it difficult to conclude whether there is a significant drop at day 8 when green female head-thoraces were analyzed. Female abdomens show significant difference between the different tested periods (*F* = 2.56, df = 7, *p* = 0.05). Tukey’s post hoc test shows a significant increase in mRNA level at 6 days after eclosion in the abdomens of the green females as compared with the brown females (*p* < 0.05) (Fig. 8B). Significant differences in *fmt*D transcript expression were observed in male head-thoraces (*F* = 3.96, df = 7, *p* = 0.011). However, Tukey’s post hoc test shows that there are no significant differences between green and brown males’ head-thoraces and abdomens at any time after eclosion (*p* > 0.05). Eight days after eclosion the expression level in brown males head-thoraces is significantly higher (*p* < 0.05) than at day 1 and 3, after eclosion (Fig. 8C). An increase in transcript expression is observed over time, which may be associated with an increase in JH titer during mating. Male abdomens show significant differences in *fmt*D transcript expression at different times after eclosion (*F = *7.13, df = 7, *p = *0.0006) (Fig. 8D), however, Tukey’s post- hoc test shows no significant difference (*p* > 0.05) between green and brown male abdomens. A significant (*p* < 0.05) increase in expression was observed between day 1, day 6, and day 8 after eclosion in green and brown abdomens indicating that JH may play a role in mating at day 1 and in egg development.

## 3 Discussion

Insecticidal control of *D. citri* that transmits *C.* Liberibacter *asiaticus* relies heavily on broad-spectrum pesticides; therefore, new approaches need to be explored to control this pest insect. JH plays an important role in many physiological processes in insects, and its biosynthetic pathway is tightly regulated [6]. Thus, identifying and characterizing enzymes that are ultimate and penultimate in the JH pathway may be good targets for effective control of these and many other important pest insects [7]. We explored the *D. citri* transcriptome and genome data to find putative penultimate/ultimate gene in the JH biosynthetic pathway of *D. citri* as was done earlier for mosquitoes [11]. As a result, a JHAMT ortholog gene (7368 bp) was identified in the *D. citri* genome. The gene codes a transcript of 819 nt, encoding a protein of 272 amino acids including a conserved glycine-rich motif strongly indicating a SAM-MT binding site (Fig. 1) and allowing proper positioning of the methyl donor SAM with its substrate for methyl ester formation (Fig. 4). This motif is found in yeast SAM-dependent methyl transferases [36] and in conserved JHAMT orthologs of *A. aegypti*, *B. mori*, *A. pisum*, *D. melanogaster*, *S. gregaria* and *T. castaneum* [11–15,25]. *D. citri* FA-o-MT exhibits sequence similarities to JHAMT orthologs in 3D folding and SAM binding site sequences. Both JHA III and FA can dock into the active groove of the 3D model that was built. Because currently there is no FA/JHA III-JHAMT X-ray-resolved complex available to use as a template our 3D model cannot predict the most optimal position of FA or JHA III in the catalytic groove (Fig. 4). In order to characterize the enzyme, and find its specificity, different substrates were tested and kinetic analyses were determined (Table 2, Fig. 5). Although several of the JH I isomers (*cis/trans/cis*; 2*Z*, 6*E*, 10*cis* and *trans/cis/cis*; 2*E*, 6*Z*, 10*cis*) bind poorly to the JH I binding protein as compared with the JH I isomer (*trans/trans/cis*; 2*E*, 6*E*, 10*cis*) [61], they are biologically active when tested on *Tenebrio* by puncture and by injections [62]. Topical application of 3 JH I mimicking isomers to *Tenebrio* pupa (0.1 μg) showed that their biological activity is 80–100%. At lower concentrations (0.01 μg/pupa) when the epoxide ring geometry is *trans* the activity dropped to 50% as compared with a *cis* geometry of the epoxide ring [63]. Recently it was shown that the JH receptor, methoprene-tolerant also binds methoprene and pyriproxyfen that do not bind the JH I binding protein because they exhibit different chirality than JH I (*trans/trans/cis*; 2*E*, 6*E*, 10*cis*). Since many juvenoids with JH activities have been identified [64,65] we tested several isomers of JHA I whose methyl ester derivatives bind poorly to the JH I receptor [61], but show biological activity [62,63]. FA-o-MT exhibits the highest affinity to homoFA, however, the methylation rate (*V_max_*) and the turnover number (*k_cat_*) of this substrate are 3.5-fold lower as compared with FA, whereas the catalytic efficiency (*k_cat_*/*K_m_*) is 6.6-fold higher than FA indicating that chirality plays an important role and methylation is not efficient when homoFA occupies the active groove as compared to FA (Fig. 4). Incubation of JHA III and FA with the enzyme shows that FA is methylated 57-fold faster than JHA III, and FA has 5.5 and 58-fold higher affinity (*K_m_*) and higher turnover number (*k_cat_*), respectively than JHA III (Table 2). In comparison, the previously characterized mosquito *Aea*JHAMT methylates JHA III 5-fold faster than FA [11]. Based on these observations, we propose that the *D. citri* enzyme that was cloned and expressed is a FA-o-MT and not JHAMT. Since no other SAM-MT like enzymes in the JH pathway were found in the genome of *D. citri* it seems that, in this insect, JH III is probably synthesized from MF and not from JHA III as was shown in mosquitoes [11].

**Fig. 6:**
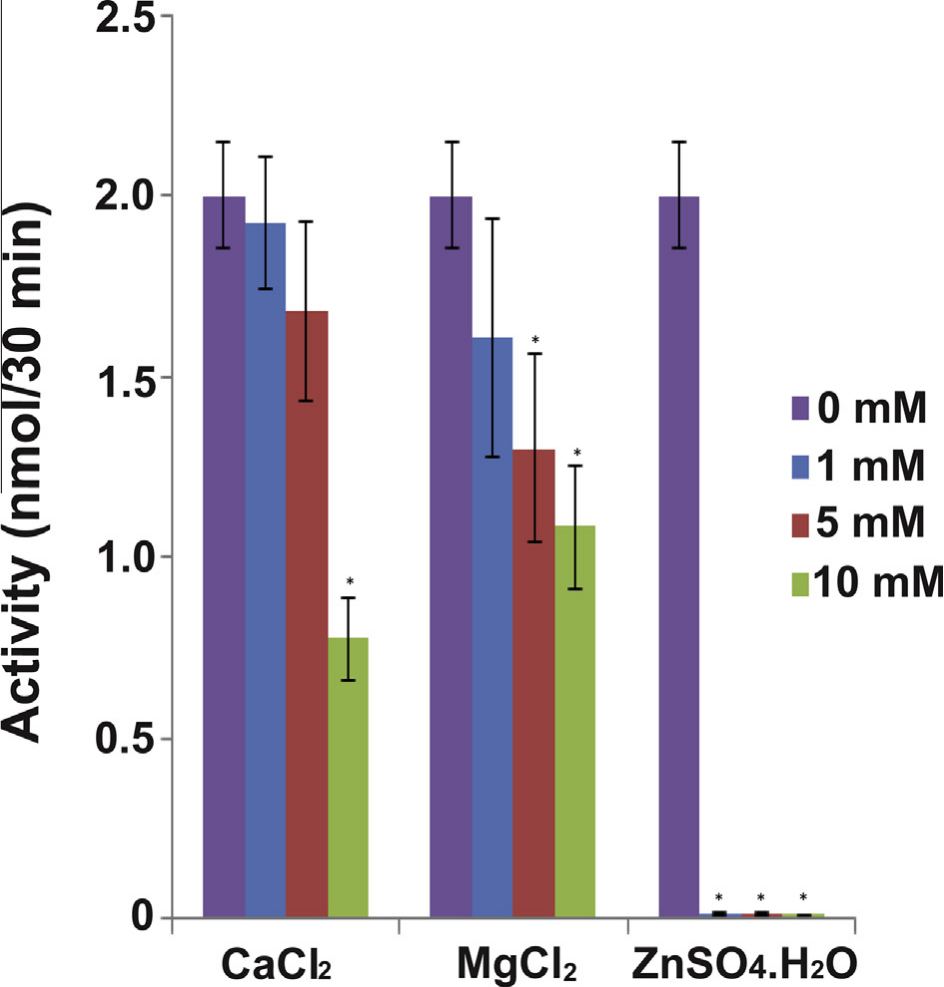
*Dc*FA-o-MT enzymatic activity in the presence of increasing concentrations of metal ions. The results are expressed as means of 3 determinations ± S.E.M. ^∗^indicates a significant difference (*p *< 0.05) as compared with the control incubated without a metal ion.

**Fig. 7:**
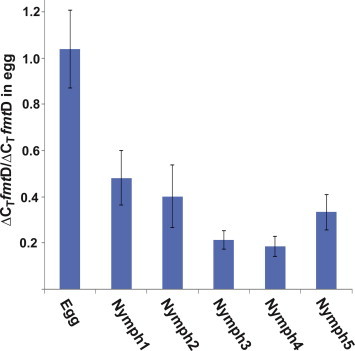
Relative *fmt*D transcript level in 24 h old egg as compared with different stages of *D. citri* nymphal developmental stages The ratio between the level of the transcript in the egg and different nymphal stages was calculated using the ΔΔ*C_T_* method [34]. In each experiment RNA (100 ng) from eggs and different nymphal stages were compared*. fmtD* transcript in the egg is significantly higher than in the nymphal stages (*p *< 0.01). No significant difference in *fmt*D transcript expression is found between the different nymphal stages.

Metal ions (Mg^2+^, Ca^2+^, and Zn^2+^) are not required for *Dc*FA-o-MT activity. However, Zn^+2^ (1 mM) reduces the enzymatic activity by 200-fold, similar to the results that were reported for *Aea*JHAMT [11]. *Dc*FA-o-MT and *Aea*JHAMT [11] have an H residue at the catalytic groove (Fig. 1B,Fig. 4), and Zn^2+^ ion strongly interact with H having a *K_d_* of 8.76 × 10^−13^ M and a relative energy of −34.26 kJ mol^−1^ blocking the catalytic site and causing inhibition (Fig. 6) [53,54]. Zn^2+^ accumulates in citrus phloem, and the amount of Zn^2+^ increases 5.6-fold (from 98 ppm to 547 ppm) after spraying citrus with zinc [55], and metal transporters are known in plants [56]. It is tempting to suggest that an increase in Zn^2+^ content of citrus phloem could inhibit *Dc*FA-o-MT and interfere with nymphal to adult development of *D. citri*.

Earlier characterization of several FA-o-MT like enzymes from Crustacea (*L. vannamei*, *H. americanus*, *C. pagurus*) and insect species (*C. capitata*, *N. lugens and M. scutellaris*, *D. melanogaster* and *S. gregaria*) reported that these FA-o-MT like enzymes did not methylate FA and are not involved in JH biosynthesis except for a report from *M. ensis* that showed low conversion of FA into MF [16–25]. These FA-o-MT-like enzyme classifications are based on protein similarity with *M. ensis* FA-o-MT, they lack a SAM-MT binding site (Fig. 1B) and do not methylate MF, or JHA. On the other hand, *D. citri* FA-o-MT exhibits a SAM-MT binding site, affinity to FA that is 5.5-fold higher than to JHA III and preferentially methylates FA. Marchal et al. [25] suggested that these proteins should not be classified as FA-o-MT-like enzymes because their biological function is yet to be determined. Thus*, Dc* FA-o-MT is the first enzyme that exhibits higher activity and affinity for FA than for JHA III with sequence similarities to many JHAMT orthologs except *S. gregaria* JHAMT which shows sequence similarity to its FA-o-MT ortholog. The latter, however, lacks SAM-MT binding site (Fig. 2) and is not involved in JH biosynthesis [25].

Earlier reports indicate that only JH III is synthesized by Hemiptera [37,38]. Later, JH I and JH III skipped bisepoxide (methyl (2R, 3S, 10R)-2,3;10,11-bisepoxyfarnesoate) were found in *R. clavatus* and *Plautia stali*, respectively [39–41]. Our results indicate that FA and homoFA are efficiently methylated by *Dc*FA-o-MT, and the only gene that is found in the genome of *D. citri* is *fmt*D. These results strongly suggest that both JH III and JH I can be synthesized from MF and homoMF, respectively by cytochrome P-450 epoxidase, and JH III in *D. citri* is not synthesized from JHA III as was shown in *A. aegypti* [11] but from MF, and FA-o-MT is the penultimate enzyme in the biosynthetic pathway of JH III (Fig. 9) in this species. *Dc*FA-o-MT is the only SAM-MT enzyme in the JH III biosynthetic pathway in *D. citri* that is found in the *D. citri* genome (http://psyllid.org/node/1). JH is inactivated by JHE in insects in order to control its titer in the hemolymph [43] and a putative JHE from *D. citri* was recently cloned and sequenced in our laboratory (accession number KM507200). Thus, JHA III can be converted back into JH III by *Dc*FA-o-MT circumventing the JH III biosynthetic pathway (Fig. 9).

**Fig. 8:**
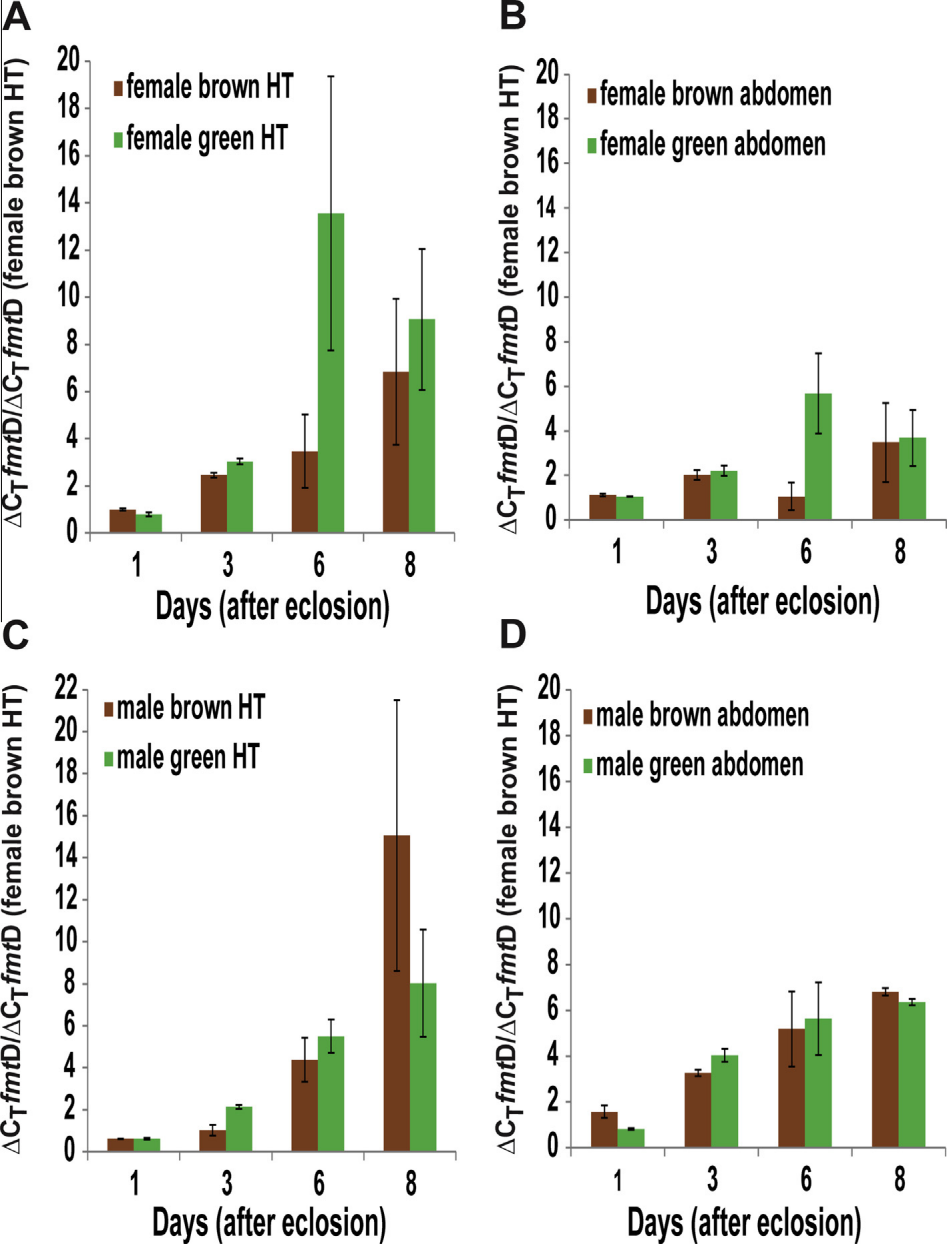
Relative *fmt*D transcript levels at 1, 3, 6, and 8 days after adult eclosion in green and brown adult *D. citri*. Fold differences in expression is calculated using the ΔΔ*C_T_* method [34], in comparison with brown female head-thorax transcript level. Extracted RNA (100 ng) was used in each assay to compare the level of *fmt*D transcript with female brown HT. (A) Comparison between transcript levels of in brown and green HT. No significant differences were observed during day 1–3, a significant increase in *fmt*D transcript expression is found at day 6 after eclosion in green female HT as compared with expression at day 1 and 3 (*p *< 0.05) and at day 8 as compared with day 1. (B) Comparison between green and brown female abdomens shows a significant increase in transcript expression at day 6 as compared with day 1, 3 and 8 (*p *< 0.05). (C) Comparison between *fmt*D transcript expression in the head-thoraces of brown and green males HT shows a significant increase in *fmt*D transcript at day 8 (*p *< 0.05) as compared with transcript level at day 1 and 3. (D) Comparison between brown and green male abdomens shows a significant increase in transcript level between day 1 and 8 after eclosion (*p *< 0.05). HT = head-thorax.

**Fig. 9:**
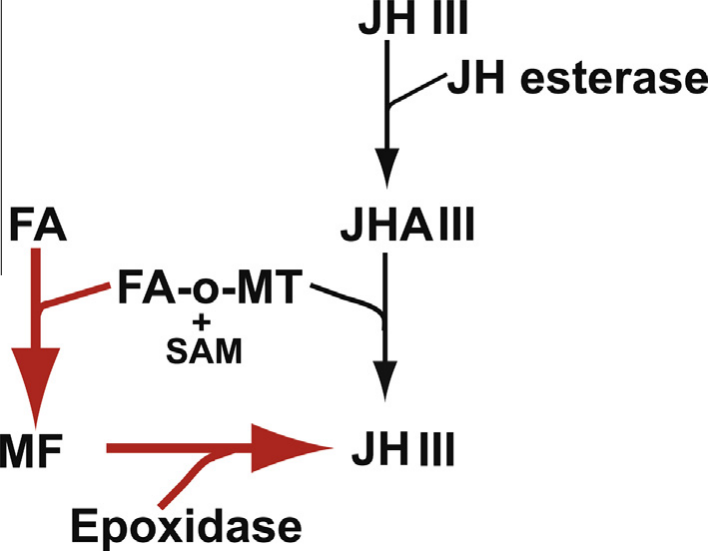
Last steps in *D. citri* JH III biosynthetic pathway. A proposed predominant pathway for JH III synthesis is shown in red arrows. *D. citri* JH III biosynthesis pathway uses FA-o-MT as the penultimate enzyme in the JH III biosynthetic pathway. MF is converted into JH III with cytochrome P-450 epoxidase (red arrows). The low level of activity on JHA III allows the enzyme to shunt back JHA III after hydrolysis of JH III by esterase (black arrows).

The *fmtD* transcript is highly expressed in newly laid eggs of *D. citri* indicating that an increase in JH titer plays a role in embryo development before the first nymphal molt as was reported for *Locusta migratoria* [42]. Following nymphal emergence the *fmt*D transcript fluctuates very little during the 5 different stages indicating that MF titers do not fluctuate much during nymphal development, however, they can still fluctuate during each nymphal stage (was not measured). Even though *fmt*D transcript is abundant during the nymphal stages, JH III titers can be controlled by cytochrome P-450 linked epoxidase that converts MF into JH III and by JHE that converts JH III into JHA III, lowering the JH titer before each molting event as was reported for the cockroach *Nauphoeta cinerea* [43]. The *fmt*D transcript is expressed during female and male *D. citri* adult life (Fig. 8). In green female head-thoraces region where the CA is located and abdomens where the ovaries are located, the transcript significantly increases six days after eclosion indicating that in this insect, as was shown in female *A. aegypti*, JH is probably required for previtellogenic oocyte development, fat body stimulation before vitellogenin biosynthesis, and to make the ovaries competent for sequestering vitellogenin [44,45]. Wenninger and Hall [46] reported that adult female *D. citri* attain reproductive maturity 2–3 days after adult eclosion, and oviposition starts 1–2 days after mating. Therefore, the increase in *fmt*D transcript during the first six days after adult-eclosion correlates with a possible increase in JH titer needed for this reproductive maturity and mating behavior. Indeed, a correlation between higher fecundity and green abdominal color of female *D. citri* was reported [47]. This report shows higher expression of *fmt*D transcript in green females at day 6 after adult eclosion that could correlate with a higher JH titer that is important for fecundity, and mating behavior of insects [9,48–50]. However, other factors like phosphorylation and post transcriptional modification may also play a role in the enzyme’s activity and cannot be ignored. Earlier reports by Borovsky et al. [51] and Van Ekert et al. [11] show that JH is synthesized by *A. aegypti* ovaries. The expression of *fmt*D transcript in the female abdomens could be correlated with JH synthesis in the ovaries as in A*. aegypti* [51,11]. In male head-thoraces and abdomens, increase in *fmt*D transcript was observed for 8 days. These results correlate with JH biosynthesis by the CA and by the male accessory glands [52]. Thus, FA-o-MT is a key enzyme in the JH III biosynthetic pathway as was reported for JHAMT orthologs of *B. mori* and *S. cynthia* [10,15].

## 4 Materials and methods

### 4.1 Chemicals

homoFA, JH I (*cis/trans/cis*; 2*Z,* 6*E,* 10*cis*), JH I (*trans/cis/cis*; 2*E,* 6*Z,* 10*cis*), and JH I bisepoxide were provided by Professor K. Sláma (Czech Republic, Prague). JH III bisepoxide [66] and FA were provided by Professor G. Prestwich (University of Utah, UT). JH I and III bisepoxides were only used to calibrate our HPLC column because the amounts that we had were too little and thus, were not hydrolyzed into the JHA bisepoxides or used to characterize FA-o-MT. JH III was purchased from Sigma (St. Louis, MO), SAM was purchased from New England Biolabs (Ipswich, MA), and [methyl-^3^H]SAM was purchased from Perkin-Elmer (Waltham, MA). All substrates used were 90 to 100% pure as shown by C_18_ RP-HPLC and by GC MS and were stored as the original solution under nitrogen at −20 °C. Under these conditions the substrates were stable for long period of time with no apparent isomerization. MF and FA are essentially the E isomers (>96% purity) [67] and the structures were confirmed by IR and proton NMR and the purity (97–98%) was confirmed by GC. Under the short experimental incubation at pH 7.9, FA and the other JHA analogues that were used are stable. homoFA, JH I (*cis/trans/cis*; 2*Z,* 6*E,* 10*cis*), JH I (*trans/cis/cis*; 2*E,* 6*Z,* 10*cis*), and JH I bisepoxide were synthesized by Dr. Albert Pfiffner (Hoffman La Roche. Switzerland) [68] and were stored as pure compounds under nitrogen at −20 °C. Under this condition very little isomerization was observed. We routinely checked the integrity of the compounds by running them on GC MS and by HPLC so the concentration of the material was always known.

### 4.2 Experimental insects

*D. citri* were reared on *Citrus macrophylla* plants in screen cages in an insectary at 25 °C with 13:11 h light: dark cycle. Every week, 200 *D. citri* were removed to freshly flushing plants, allowed to lay eggs for 7 days and then removed from the plant leaves by aspiration. Nymphs and eggs were collected by brushing them off the flush with a small paint brush or by gently removing them with the tip of a pin. Insects that were used for RNA isolation were stored at -80 °C.

### 4.3 RNA preparation

RNA was extracted at different developmental life stages of *D. citri* in TRIzol® (Life Technologies, Carlsbad, NY) following a modified manufacturer protocol [28]. Briefly, adults, eggs, and nymphs were separately transferred into tubes containing acid-washed glass beads (180 μm) (Sigma). TRIzol was added and the samples were incubated for 5 min at room temperature. Tissues were broken in a FastPrep instrument for 40 s at speed 6 m/s (meter/s), chloroform added, and the samples incubated for 10 min at room temperature. Tissues were pelleted by centrifugation (13,400*g* for 10 min at 4 °C) and the aqueous phase transferred to a fresh tube. Isopropanol was added and the samples were incubated at room temperature for 10 min and then centrifuged (13,400*g* for 8 min at 4 °C). The pellet was washed with 75% ethanol and the samples centrifuged (6900*g* for 5 min at 4 °C). The ethanol was removed and the RNA pellet air-dried for 10 min. The pellets were dissolved in water, and RQ1 DNase and DNase buffer (Promega, Madison, WI) were added and the samples incubated at 37 °C for 1 h. After incubation, water and phenol (pH 4.3) were added and the samples centrifuged (13,400*g* for 6 min at 4 °C), the upper phase transferred to a fresh tube and an equal volume of phenol/chloroform was added. The samples were centrifuged and the upper phase transferred to a fresh tube and precipitated in sodium acetate (3 M, pH 5.2) and 100% ethanol at −20 °C overnight. The pellets were centrifuged (13,400*g* for 15 min at 4 °C) and then washed in 75% ice cold ethanol, re-centrifuged (13,400*g* for 8 min at 4 °C) and the ethanol removed and the pellet, air dried and suspended in water. RNA concentrations were determined at A_260_ and A_280_ in a NanoDrop 1000 instrument (Thermo Fisher Scientific, Waltham, DE), and stored at −80 °C.

### 4.4 cDNA cloning and sequencing

A full length cDNA was sequenced in two stages by first identifying a short 3’ end sequence of *D. citri* FA-o-MT gene (*fmt*D) in EST and cDNA libraries of *D. citri* (http://www.sohomoptera.org), by blastn searching with JHAMT ortholog nucleotide sequences from *A. aegypti*, *A. pisum*, *D. melanogaster*, and *B. mori* (GenBank accession numbers: DQ409061.1, NM_001162779, NM_135949.2, NM_001043436, respectively). To clone the short length *fmt*D sequence, RNA was extracted from 30 *D. citri* adults a week after adult eclosion, reverse transcribed using reverse primer B: 5′-TCA TTT CCT GGC GAA CAC AAT-3′, *t_m_* 50.5 °C, (Table 1), at the 3′ end sequence of *fmt*D, in a reaction mixture (20 μL) containing 4 μL 25 mM MgCl_2_, 2 μL 10 × PCR buffer (Applied Biosystems, Foster City, CA), 6 μL sterile distilled water, 4 μL dNTP-mix (10 mM each of dATP, dTTP, dCTP, and dGTP), 1 μL RNase inhibitor (20 U), 1 μL Moloney murine leukemia virus (MMLV) reverse transcriptase (50 U), 1 μL reverse primer B (10 μM), and RNA (1 μg). Reverse transcription was performed in a DNA thermal cycler (PTC-100, BioRad, Hercules, CA) at 24 °C for 10 min, followed by 42 °C for 60 min, 52 °C for 30 min, 99 °C for 5 min, and 5 °C for 5 min. After RT, 3 μL 10 × PCR buffer, 25.5 μL sterile distilled water, 0.5 μL AmpliTaq DNA polymerase (2.5 U), and 1 μL (10 μM) of forward primer A: (5′-TCT TCA CTG GGT TCA GGA TCA GA-3′ (*t_m_* 53.0 °C), (Table 1) were added to each reaction tube. PCR was carried out as follows: denaturation for 3 min at 95 °C, annealing for 4 min at 48 °C, extension for 40 min at 60 °C (one cycle each), denaturation at 95 °C for 30 s, annealing for 30 s at 48 °C, and extension for 2 min at 60 °C (40 cycles) with a final extension for 15 min at 60 °C. Following PCR, the dsDNA was separated by agarose gel (2%) electrophoresis in the presence of ethidium bromide and a DNA amplified band (481 bp) was visualized under UV. The DNA band was cut from the gel, and eluted using NucleoSpin Gel and PCR Clean-up kit (Macherey–Nagel, Bethlehem, PA) and cloned into pCR2.1 following manufacturer’s instructions (Invitrogen, Carlsbad, CA). INVαF’ *E. coli* cells (allowing stable replication of high copy number plasmids and blue/white screening of recombinant clones) were transformed and grown overnight on LB plates in the presence of kanamycin (50 μg/mL). Colonies were picked from the plates and grown in LB medium in the presence of kanamycin (50 μg/mL) and plasmids were purified from each culture using a QIAprep Spin Miniprep Kit (Qiagen, Germantown, MD). Plasmids were sequenced at the Genomics Core of the USDA-ARS USHRL in Fort Pierce, FL, confirming the *fmt*D cDNA (481 bp) sequence. To obtain a full length *fmt*D cDNA, a ClustalW alignment was done using *A. pisum* JHAMT cDNA sequence on *D. citri* genome (http://psyllid.org/node/1) identifying the 5′ sequence of *fmt*D cDNA including the start codon. A gene-specific primer C: 5′-ATG CAT AAA GCG ACC CTA TAC-3′ (*tm* 50.5 °C) (Table 1) was then synthesized and *D. citri* RNA was reverse transcribed using reverse primer B (as described above). The ssDNA was amplified by PCR using forward primer C as described above. After PCR, a dsDNA (819 bp) was separated by agarose gel (2%) electrophoresis, visualized under UV, cut from the gel, and purified as described above. The dsDNA (819 bp) was cloned into pCR2.1, INVαF’ *E. coli* cells were transformed, and plasmids were purified and sequenced, confirming a full-length sequence similar to the transcriptome EST sequences and the genomic sequences (http://psyllid.org/node/1). The full length sequence was then deposited in GenBank (accession number KM212828) and *fmt*D ORF was blasted against the available *D. citri* genomic data (http://psyllid.org/download) to identify exon and intron sequences.

### 4.5 Molecular modeling of *Dc*FA-o-MT

Using *fmt*D ORF sequence, a homology model was built using the YASARA structure program to determine if *Dc*FA-o-MT folds similar to models of insect SAM-MTs. Several *Dc*FA-o-MT models were built using templates from X-ray coordinates of methyltransferase of *Anabæna variabilis* (Bacteria) (PDB 3CCF) (to be published by the Joint Center of Structural Genomics, DOI: 10-2210/pdb3ccf/pdb), the trans-aconitase-3-methyltransferase of yeast (PDB 3G5T), and the dimethyladenosine transferase of *Plasmodium falciparum* (protozoan parasite) (PDB 2H1R) [29]. A hybrid model was then constructed from the three previous models. PROCHECK [30] and ANOLEA [31] were used to assess the geometric quality and the energy of the three-dimensional model (3D). Docking of FA, JHA III, and SAM was performed with the YASARA structure program. Molecular models were drawn with YASARA and Chimera [32].

### 4.6 *Dc*FA-o-MT Expression in bacterial cells

*Dc*FA-o-MT was cloned into the expression vector Champion™ pET200 Directional TOPO® (pET200/D-TOPO) (Invitrogen). Cloning was accomplished using forward primer D: 5′-*cacc*ATGCATAAAGCGACCCTATAC-3′ (*t_m_* 57.7 °C), (Table 1) using TOPO cloning. Full-length *Dc*FA-o-MT, including *cacc* at the 5’ end, was amplified by PCR using a Phusion High-Fidelity PCR kit (Thermo Fisher Scientific). The PCR reaction (20 μL) contained, 4 μL 5 × HF buffer, 13.4 μL sterile distilled water, 200 μM of each dNTP, 0.4 U Phusion DNA polymerase, 0.5 μM each of the forward and reverse primers (D and B, respectively) (Table 1), and 1 ng template DNA of *fmt*D ORF cloned into pCR2.1 (as above). Initial denaturation was carried out at 98 °C for 30 s followed by 35 cycles of denaturation at 98 °C for 10 s, annealing at 66 °C for 30 s, extension at 72 °C for 20 s and a final extension at 72 °C for 10 min. After PCR, a 823 bp dsDNA was separated by agarose gel (2%) electrophoresis, visualized by UV light, cut and eluted from the gel and topo-cloned into pET200/D-TOPO following the manufacturer’s protocol. The vector was sequenced and the *Dc*FA-o-MT sequence confirmed.

*E. coli* one Shot BL21 (DE3) competent cells were transformed with pET200/D-TOPO carrying *fmt*D ORF. Cells were grown overnight at 37 °C in LB medium (5 mL) in the presence of kanamycin (50 μg/mL). After incubation, the cells were centrifuged at 1500*g* at 4 °C for 10 min and the pellet resuspended in LB medium (500 mL) containing ampicillin (50 μg/mL). The resuspended cells were grown at 37 °C to an OD_600_ of 0.6 (∼6 × 10^8^ cells). Transcription and synthesis of *Dc*FA-o-MT was initiated by adding IPTG (1 mM) (Gold Biotechnology, St. Louis, MO) and the cells were incubated overnight at room temperature, shaking at 225 rpm. After the induction period, the BL21(DE3) cells were incubated with a bacterial protein extraction reagent (B-PER, Pierce, Rockford, IL) in the presence of a bacterial protease inhibitor cocktail (Sigma) in a shaker for 20 min at room temperature. After incubation, glass beads (180 μm) were added and the cells were broken in a FastPrep Instrument for 40 s at a speed of 4 m/s (meter/s), centrifuged at 12,800*g* for 10 min at 4 °C, and the supernatant stored in a 5% glycerol solution containing 20 mM β–ME (Sigma) at −20 °C. The extracted histidine-tagged *Dc*FA-o-MT was adsorbed onto a nickel column (Ni–NTA, Qiagen) (0.5 × 5 cm; DxH), containing Ni–NTA (5 mL) and equilibrated with a wash buffer (50 mM NaH_2_PO_4_, 300 mM NaCl, pH 8.0). The column was washed with 20 mL of the same buffer at a speed of 0.2 mL/min followed by 15 mL consecutive washes of wash buffer containing 20 mM, 40 mM, and 60 mM imidazole. *Dc*FA-o-MT was eluted from the column with 250 mM imidazole and fractions (1 mL) were collected. Protein concentrations were determined using a Bradford protein assay (BioRad). To prevent loss of enzyme activity during the Ni affinity column purification, the wash buffer contained 10% glycerol and 20 mM β–ME. To maintain enzymatic activity, with no loss for several months, glycerol was added to a final concentration of 50%, and aliquots (200 μL) were stored at −20 °C. Column fractions after the Ni affinity chromatography were analyzed by SDS PAGE (NuPage 4–12% Bis Tris, Life Technologies) and stained with Coomassie brilliant blue (BioRad).

### 4.7 Substrate preparation and C_18_ RP-HPLC analysis

Substrates (JHA I (*cis,trans*, *cis*; 2*Z*, 6*E*, 10*cis*) JHA I (*trans/cis/cis*; 2*E*, 6*Z*, 10*cis*) and JHA III) were prepared from their methyl esters and purified by C_18_ reversed phase (RP)-HPLC as described by Van Ekert et al. [11] and Borovsky and Carlson [33]. Briefly, JH substrates were incubated with 0.5 N NaOH in ethanol for 24 h at room temperature and the JHAs were purified by C_18_ RP-HPLC. The acetonitrile was evaporated under a gentle stream of nitrogen and the aqueous solution extracted two times in hexane, the hexane was then evaporated with nitrogen and the JHAs dissolved in DMSO. Stock solutions for each acid substrate (100 μM) in DMSO were stored at −20 °C under nitrogen at pH 8.0 until used. To avoid long storage of the JHA substrates each substrate was prepared only when needed for the kinetic characterization studies. A Spectra-Physics P2000 RP-HPLC system was used for the separation of the methyl esters, using a linear acetonitrile water gradient (40–100%) [33], a C_18_ RP column (100 Å, 150 mm × 4.6 mm, 5 μm particle size, Varian Microsob-MV), a spectromonitor with UV detection at 214 nm (LDC/Milton Roy Spectromonitor), and an integrator. The C_18_ RP column was calibrated with non-radioactively labeled standards (4 μg, each) of MF, homoMF, JH III, JH I, JH III bisepoxide, and JH I bisepoxide eluting at 33, 38, 20, 25, 13, and 14 min, respectively [33].

### 4.8 Activity measurement and determination of kinetic parameters

Recombinant *Dc*FA-o-MT (357 pmol) was incubated in separate reaction mixtures (100 μL) containing FA, homoFA, JHA I (*cis,trans*, *cis*; 2*Z*, 6*E*, 10*cis*) JHA I (*trans/cis/cis*; 2*E*, 6*Z*, 10*cis*) and JHA III (2*E*, 6*E*, 10*cis*) (1–20 μM, each), 50 mM Tris–HCl buffer (pH 7.9), 0.55 μCi [3H] SAM (68 nM) and non-radioactively labeled SAM (10 μM, saturating concentration) for 45 min at 25 °C. After incubation, acetonitrile (100 μL) containing MF (85.1 nM) and, JH III (250 nM) was added. The conversion of the substrates into [3H] labeled methyl esters was followed by C_18_ RP-HPLC, rapid biphasic separation (RBS) and [3H] labeled samples analyzed by liquid scintillation [11]. Substrate methylations and linearity were followed by incubations at different time intervals (0, 1, 5, 10, 20, 30, 45, and 60 min) in triplicate and the results analyzed by linear regression using GraphPad Prism (La Jolla, CA). The saturating concentration of SAM for Michaelis Menten kinetic analyses were determined by incubating substrates with increasing concentrations of non-radioactively labeled SAM (0.1–640 μM) [11]. Michaelis Menten kinetic parameters (*K_m_*_,_ *V_max_* and *k_cat_*) were determined in reaction mixtures (as above) in the presence of 10 μM SAM (saturating concentration), *Dc*FA-o-MT (357 pmol), and increasing concentrations (0–20 μM) of FA, homoFA, JHA I (*cis/trans/cis*; 2*Z*, 6*E*, 10*cis*) JHA I (*trans/cis/cis*; 2*E*, 6*Z*, 10*cis*) and JHA III (2*E*, 6*E*, 10*cis*) [11]. Results are expressed as means of 3 determinations ± S.E.M. Reaction rates were plotted against concentrations and Michaelis Menten kinetic parameters, *V_max_*, *K_m_*, and *k_cat_* were calculated from Lineweaver–Burke double reciprocal plots using GraphPad Prism.

### 4.9 Measurement of metal dependency

Metal ion dependency of *Dc*FA-o-MT, was followed using increasing concentrations (1–10 mM) of CaCl_2_, MgCl_2_, or ZnSO_4_.H_2_O in reaction mixtures containing 50 mM Tris–HCl buffer (pH 7.9), 0.55 μCi [3H] SAM (68 nM), SAM (10 μM), *Dc*FA-o-MT (357 pmol), and 5 μM of substrate for 30 min at 25 °C [11]. The results are expressed as means of 3 determinations ± S.E.M.

### 4.10 Analysis of *fmt*D transcript by RT-qPCR

The level of *fmt*D transcript during different developmental stages and in different tissues was followed by RT-qPCR. Total RNA was extracted in TRIzol (as above) from eggs, different stages, head-thoraces and abdomens of green and brown color variants of female and male adults to find out if there was a difference in JH biosynthesis between the two variants. Each sample was collected from 3 different groups and RT-qPCR analyses were done on extracted RNA (100 ng) using the QuantiTect SYBR Green RT-PCR kit (Qiagen). Each RT-qPCR reaction was run in duplicate including a control reaction without reverse transcriptase to check for DNA contamination. Primers, E: 5′-ATCTCGCCATACCACCACTC-3′ (*t_m_* 49.5) and F: 5′-CCTCACTGTCTGCTCATCCA-3′ (*t_m_* 53.8 °C) (Table 1) were used to amplify a fragment (307 bp) with an efficiency of 1.74 ± 0.01 (Rotor-Gene 6000 series software 1.7). An ANOVA and Tukey’s post hoc test showed no significant difference in efficiency among the tested samples (*F *= 1.17, df = 23, *p *= 0.31). Analyses of the melt-curve profiles, showed a single peak and the reaction products were analyzed by agarose gel (2%) electrophoresis and the sequence verified. Gene expression was normalized with the expression of a constitutive gene of *D. citri*, glyceraldehyde 3-phosphate dehydrogenase (GAPDH) that is expressed in all life stages studied [57]. GAPDH primer pair (forward: 5′-TCAACGGTTTCGGACGTATT-3′ (*t_m_* 49.7 °C) and reverse: 5′-CCGTTCACAACCAGGAAGTT-3′ (*t_m_* 51.8 °C)) (Table 1) was used exhibiting a *C_T_* of 13.19 ± 0.01 (*F *= 1.76, df = 15, *p *= 0.09) and efficiency of 1.74 ± 0.01 that was not significantly different from the tested samples (*F *= 0.79, df = 23, *p *= 0.73). After RT-qPCR, a single melt peak was observed and the product was verified by sequencing. The gene expression of the target gene (*fmt*D) was quantified using the ΔΔ*C_T_* method [34]. To compare freshly laid eggs (24 h old), and different nymphal stages the *fmt*D transcript level in the laid eggs was used to calculate fold ratio differences in comparison to different nymphal stages. Since male and female *D. citri* adults exhibit different body colors of brown and green, the brown female head-thorax *fmt*D transcript level at day 1 after adult eclosion was used to calculate fold ratio differences. The variance among the groups was analyzed by ANOVA (*α* = 0.05), and a Tukey’s post hoc test (*α* = 0.05) to determine significant differences between samples.

### 4.11 Statistical analysis

Data were analyzed by the student’s *t*-test and by Kruskal–Wallis (non-parametric) ANOVA and Tukey’s post hoc test using JMP statistical package version 4 (SAS Institute Inc., Cary, NC) and GraphPad Prism v5.0. All lines for *K_m_* analyses were drawn using linear regressions (*r*^2^ > 0.99) containing at least 7–10 data points. Results were considered statistically significant when *p *< 0.05 and results are expressed as means of 3 determinations ± S.E.M., except where otherwise stated.

## Author contribution statement

E.V.E. preformed the experiments, was involved in the experimental design, analyzed the data and wrote the paper, R.G.S. Jr. analyzed the data, and was involved in the experimental design. P.R. analyzed the data and built the 3D model, C.A.P. analyzed the data and was involved in the experimental design, G.S. analyzed the data, D.B. analyzed the data, was involved in the experimental design and wrote the paper.

## Figures and Tables

**Table 1:** Primers used for cloning of *fmt*D and in RT-qPCR analyses of transcript expression during different developmental stages of *D. citri*.

Name	5′–3′ sequence	*t_m_* (°C)
Primer A	TCTTCACTGGGTTCAGGATCAA	53.0
Primer B	TCATTTCCTGGCGAACACAAT	50.5
Primer C	ATGCATAAAGCGACCCTATAC	50.5
Primer D	ccacATGCATAAAGCGACCCTATAC	57.7
Primer E	ATCTCGCCATACCACCACTC	49.5
Primer F	CCTCACTGTCTGCTCATCCA	53.8
GAPDH forward	TCAACGGTTTCGGACGTATT	49.7
GAPDH reverse	CCGTTCACAACCAGGAAGTT	51.8

**Table 2:** Michaelis Menten and kinetic constants for *Dc*FA-o-MT. Enzyme assays were carried out using purified recombinant *Dc*FA-o-MT (357 pmol), 1–20 μM substrates, and 10 μM of S-adenosyl methionine. Incubation was at 25 °C for 30 min and are expressed as means of 3 determinations ± S.E.M. *K_cat_* is expressed as mol/mol enzyme.

Substrates	*K_m_* (μM)	*V_max_* (pmol/min)	*k_cat_* (s^−1^ × 10^−3^)	*k_cat_/K_m_* (s^−1^ M^−1^)
FA	0.46 ± 0.05	16.10 ± 0.57	0.75 ± 0.03	1645
homoFA	0.020 ± 0.01	4.64 ± 0.17	0.22 ± 0.01	10850
JHA I (2*Z,* 6*E*, 10*cis*)	5.65 ± 0.83	1.75 ± 0.15	0.08 ± 0.01	143.4
JHA III (2*E*, 6*E*, 10*cis*)	2.53 ± 0.72	0.28 ± 0.04	0.013 ± 0.002	5.14
JHA I (2*E*, 6*Z*, 10*cis*)	4.58 ± 4.82	0.05 ± 0.03	0.003 ± 0.001	0.66
